# Decentration following femtosecond laser small incision lenticule extraction (SMILE) in eyes with high astigmatism and its impact on visual quality

**DOI:** 10.1186/s12886-019-1153-7

**Published:** 2019-07-17

**Authors:** Jia Huang, Xingtao Zhou, Yishan Qian

**Affiliations:** 1 Department of Ophthalmology, EYE & ENT Hospital of Fudan University, Shanghai, People’s Republic of China; 2 NHC Key Laboratory of Myopia (Fudan University), Shanghai, People’s Republic of China; 3 Shanghai Research Center of Ophthalmology and Optometry, Shanghai, People’s Republic of China

**Keywords:** Femtosecond laser small incision lenticule extraction, Astigmatism, Decentration, Corneal topography

## Abstract

**Background:**

To measure the decentration following SMILE in eyes with high myopic astigmatism and investigate its impact on visual quality.

**Methods:**

The prospective study was conducted to analyze patients who underwent SMILE for correction of myopia and myopic astigmatism ≥2.5D (high astigmatism group, HA) at the ophthalmology department, Eye and ENT hospital, Shanghai, China.. Patients with myopic astigmatism < 1.5D served as controls (low astigmatism group, LA). Decentration was measured using a Scheimpflug camera with a difference map of the tangential curvature at 12 months postoperatively. Also the associations between decentration from the coaxial sighted corneal light reflex (CSCLR) and the visual outcomes, correction efficacy of astigmatism, wavefront aberrations and objective scatter index (OSI) were analyzed.

**Results:**

No significant differences were observed in the decentered distance between HA and LA in either eyes (OD: HA: 0.18 ± 0.10 mm, LA: 0.20 ± 0.14 mm, *P* = 0.659; OS: HA: 0.22 ± 0.11 mm, LA: 0.20 ± 0.11 mm, *P* = 0.637). The analysis across the three levels of decentration (< 0.1 mm, 0.1–0.2 mm, and > 0.2 mm) showed no significant association between decentration and visual outcomes of predictability, efficiency, safety, MTF cutoff, OSI, SR and OVs in both groups. Also no significant association was observed between decentration and postoperative astigmatism in either group. A significant relationship between the magnitude of decentration and induced coma and spherical aberration was observed in HA.

**Conclusions:**

The amount of decentration between HA and LA groups showed no differences. Decentration > 0.20 mm from CSCLR resulted in greater induction of coma and SA after SMILE in eyes with HA.

## Background

Small-incision lenticule extraction (SMILE) is an all-in-one procedure, in which a femtosecond lasers were used to perform intrastromal lenticule creation that is manually extracted through a small side incision. SMILE technology exhibited excellent efficacy, safety, stability and predictability in the correction of both myopia as well as myopic astigmatism [1–4]. One potential limitation of SMILE is that no active eye tracker is used during the scanning procedure. It might probably increase the risk of decentration and the visual outcomes might be influenced by the surgeon’s operative experience and patient’s cooperation. Several studies on the effects of decentration and its effects on visual outcomes have been reported till date [5–8]. Better refractive outcomes were achieved when the lenticule center was closer to the corneal vertex. In eyes with significant corneal astigmatism, shifting of the eye may occur due to uneven pressure on the cornea during suction, leading to decentration of the lenticule. Chan et al. found a significant correlation between anterior keratometric astigmatism and decentration distance in eyes following SMILE surgery, and the preoperative cylindrical error was 1.0 diopters [9]. Currently, there are few studies that investigated the effects of high astigmatism (HA) on decentration during SMILE surgery.

Hence, we conducted a prospective study to determine the characteristics of lenticular decentration following SMILE in eyes with high astigmatism (HA) and to investigate the relationship between decentration and visual outcomes.

## Methods

### Patients

This prospective study included patients who underwent SMILE for the correction of myopia and myopic astigmatism at the Ophthalmology Department of the Eye and ENT Hospital in Shanghai, China from February 2016 to January 2017. Inclusion criteria were as follows: spherical refraction from − 3 to − 10 D, astigmatism from − 2.5 to − 5 D, the best corrected distance visual acuity (BCVA) of 20/25 or better, stable refraction for 2 years prior to surgery, and the absence of other pathologic ocular conditions or relevant systemic diseases. Patients with myopic astigmatism of lower than 1.5D and a spherical equivalent (SE) comparable to those of HA group serves as controls (high astigmatism group: HA; low astigmatism group: LA).

### Surgical technique

All SMILE procedures were performed by the same surgeon using a VisuMax femtosecond laser system (Carl Zeiss Meditec AG, Jena, Germany) according to the surgical procedure described by Sekundo [6], with a repetition rate of 500 kHz and a pulse energy of 140 nJ.

The patient’s eye was positioned under an illuminated and curved suction cone after a standard sterile draping and insertion of the eye speculum. Next, the patients were asked to fixate on the internal target light, which was mounted coaxial with the femtosecond laser beam. Then the centration on the coaxial sighted corneal light reflex (CSCLR) was attempted and accepted by the surgeon when the ring of the watermark (i.e., the touch zone produced by the contact between the cornea and the cone) was concentric with the margin of the suction cone [5]. The diameter of the lenticule was 6.5 mm and the diameter of the cap was 7.5 mm. The thickness of the cap was 120um. A single side cut of 90 degrees with a circumferential length of 2 mm was made in the superior position. Following the cutting procedure, the lenticule was seperated and removed from side cut incision. No intra-operative or postoperative complications were observed. Correction was based on the preoperative manifest refraction, and all eyes were targeted for emmetropia. This study followed the tenets of the Declaration of Helsinki and was approved by the ethics committee of the EENT Hospital of Fudan University. Written informed consents were obtained from all subjects.

### Measurements of decentration

Patients were examined preoperatively and at 1, 6 and 12 months postoperatively. Objective and subjective refraction tests were performed, and the uncorrected and the corrected distance visual acuities were recorded during all the follow-up visits. Pentacam (Oculus GmbH, Wetzlar, Germany) imaging of the anterior surface was performed by the same experienced examiner, and three measurements were averaged to obtain each result. As the attempted treatment center is the CSCLR, the goal was to achieve the displacement of the lens center relative to the preoperative CSCLR. Pentacam centers its measurement on the intercept of the instrument’s optical axis and cornea, and so the point remains to be the corneal vertex. In myopic eyes with small angle Kappa, the corneal vertex will be the CSCLR. The centration of the lenticule was located as described by Reinstein et al. in his study [7]. A difference map based on the tangential curvature was generated for each eye using the preoperative and 12-month postoperative scans. The optical zone was defined on the tangential difference map as the central zone till the mid-peripheral power inflection point. The corneal vertex (or CSCLR) was displayed as the (0, 0) point and a coordinate (x, y) in millimeters could be shown for any point where the cursor moved. The observer placed a transparency with multiple concentric circles on top of the difference map, which was magnified on the screen and made as large as possible so that the center of the cutting zone (× 3, y3) could be located. The decentration (△x, △y) was calculated using the formula: △x = × 1-× 2 + × 3; △y = y1-y2 + y3, where × 1, y1 represent the preoperative pupil center coordinate and × 2,y2 represent the postoperative pupil center coordinate. The coordinate △x refers to the horizontal decentered displacement relative to the preoperative CSCLR, and △y refers to the vertical decentered displacement relative to the preoperative CSCLR. The magnitude of the decentered displacement was √(△× ^2^ + △y^2^). The displacement between the preoperative CSCLR and the pupil center is termed as chord mu instead of kappa angle according to chang et al. [10], and calculated as √(x_1_^2^+ x_2_^2^). The distribution of pupil centers with respect to CSCLR in each quadrant was plotted and compared (SN = superior nasal; ST = superior temporal; IN = inferior nasal; IT = inferior temporal).

### Wavefront aberrations and intraocular scattering measurements

The ocular wavefront aberrations were measured using an aberrometer (Wavefront Supported Custom Ablation; Carl Zeiss Meditec, Jena, Germany) and analyzed for a standardized pupil diameter of 6 mm. The Zernike coefficients of vertical coma (Z_3_^− 1^), horizontal coma (Z_3_^1^) and spherical aberration (SA) (|Z_4_^0^|) were recorded. The root mean square (RMS), that was expressed as coma ($\sqrt{{\left({Z}_3^{-1}\right)}^2+{\left({Z}_3^1\right)}^2}$), SA and higher-order aberrations (HOAs) were analyzed because of their clinical significance in determining the visual quality. Associations between the aberrations and decentrations were also assessed.

The optical quality and intraocular scattering were measured using an optical quality analysis system (OQAS™II, Visiometrics, Terrassa, Spain) with an artificial pupil diameter of 4.0 mm in mesopic conditions. We used a modulation transfer function (MTF) cutoff for evaluating the optical quality and objective scatter index (OSI) for intraocular scattering. MTF cutoff frequency represents spatial frequency at which MTF value was 0.01. The cutoff frequency was usually 30 cpd that corresponds to a 20/20 visual acuity, and the maximum MTF cutoff was not more than 60 cpd. The OSI was provided by the double-pass system and computed as the ratio of the amount of light of the peripheral zone (an annular area of 12 and 20 min) to the central zone (1 min arc central peak) of the retinal image. OSI values of ≤1.0 indicated lower scattering in the adults’ eyes [11, 12]. The ratio between the two areas under the MTF profile is Strehl ratio (SR). In other words, the ideal aberration-free eye to the aberrated eye that ranged between 1.0 and 0, and the larger SR indicates higher optical quality. Three OQAS values (OVs) at 100, 20, and 9% contrasts are calculated from the spatial frequencies corresponding to 0.01, 0.05, and 0.1 MTFs, respectively.

### Data analysis

Data analyses were performed by using statistical analysis software (PASW 18.0, SPSS Inc., Chicago, IL). Continuous variables were described as means±SD. Predictability was defined as the proportion of eyes achieving a postoperative spherical equivalent (SE) within ±0.50 Diopters (D) and ± 1.00D of the intended target. Efficacy was defined as the proportion of eyes achieving an UDVA of 20/20 and 20/40 or better postoperatively. Safety was defined as the proportion of eyes that lost or gained 1 or more lines of postoperative CDVA relative to preoperative CDVA. Considering the potential correlations between the right and left eyes of the same patients, a linear mixed regression was used to analyze the differences in decentration between high and low astigmatism groups and to compare the differences between the right and left eyes. The association between decentration and visual outcomes was also examined by linear mixed regression. The correcting efficacy of astigmatism was assessed using the correction index (CI) and index of success (IOS). CI was defined as the ratio of surgically-induced change in the astigmatism (SIA) to target-induced astigmatism (TIA). IOS was defined as the ratio of postoperative manifest astigmatism to TIA. The statistical significance level was set at 0.05.

## Results

A total of 48 eyes with astigmatism higher than 2.5 D were included in this study (HA group, mean cylinder: -3.15 ± 0.62 D, range: -5.0~ − 2.5D) and 66 eyes with astigmatism lower than 1.5D (LA, mean cylinder: -0.61 ± 0.43 D, range: − 1.5~0D) served as controls. The mean preoperative spherical error was higher in the LA group (− 3.86 ± 1.55 for HA and − 5.01 ± 1.25 for LA, *P* < 0.01) and showed no significant differences in the preoperative SE between the two groups (− 5.44 ± 1.48 for HA and − 5.31 ± 1.32 for LA, *P* = 0.642). No significant difference was found in the postoperative spherical error (0.19 ± 0.41 for HA and 0.10 ± 0.28 for LA, *P* = 0.197) or the SE (− 0.10 ± 0.41 for HA and − 0.01 ± 0.27 for LA, *P* = 0.182) between the two groups. The postoperative cylinder was significantly greater in the HA group (− 0.58 ± 0.39 for HA and − 0.21 ± 0.21 for LA, *P* < 0.01).

### Optical zone center locations

#### Preoperative chord mu

**Fig. 1:**
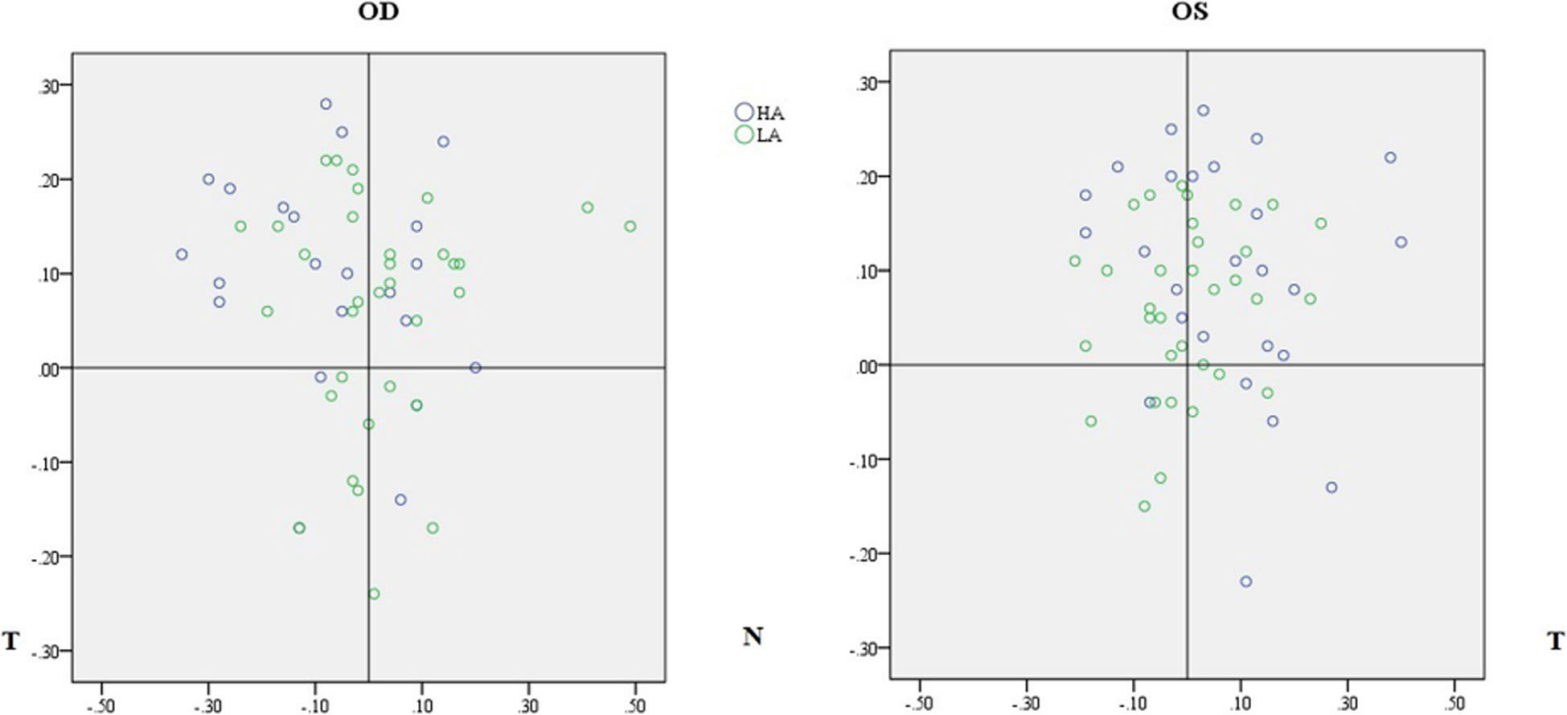
Scatter plot showing the distribution of pupil centers with respect to CSCLR in the HA and LA groups for the right and left eyes

The mean Chord mu was greater in the HA group than in the LA group (**HA:** 0.20 ± 0.09 mm, LA: 0.16 ± 0.09 mm, *P* = 0.006). A significant difference was observed in the left eyes **(OD: HA:** 0.20 ± 0.09 mm, LA: 0.17 ± 0.10 mm, *P* = 0.283; **OS: HA:** 0.20 ± 0.10 mm, LA: 0.14 ± 0.07 mm, *P* = 0.003, Fig. 1). No significant difference was observed in the magnitude of displacement between the two eyes (OD: 0.19 ± 0.10 mm, OS: 0.17 ± 0.09 mm, *P* = 0.133). In the right eyes, the pupil centers of 40 eyes (72.7%) were superior to the CSCLR and the pupil centers of 31 eyes (56.3%) were temporal to the CSCLR [SN: 17 (30.9%); ST: 23 (41.8%); IT: 8 (14.5%); IN: 7 (12.7%)]. In the left eyes, the pupil centers of 45 eyes (76.3%) were superior to the CSCLR and the pupil centers of 30 eyes (50.9%) were temporal to the CSCLR [SN: 23 (39.0%); ST: 22 (37.3%); IT:8 (13.6%); IN: 6 (10.2%)]. No significant difference was found in the distribution of pupil centers with respect to CSCLR between HA and LA groups in either of the eyes (OD: Mann-Whitney U, *P* = 0.689; OS: Mann-Whitney U, *P* = 0.758, Fig. 1). No significant correlation was found between the magnitude of Chord mu and the wavefront aberration, MTF cutoff and OSI in both the groups.

#### Decentration of treatment centers

**Fig. 2:**
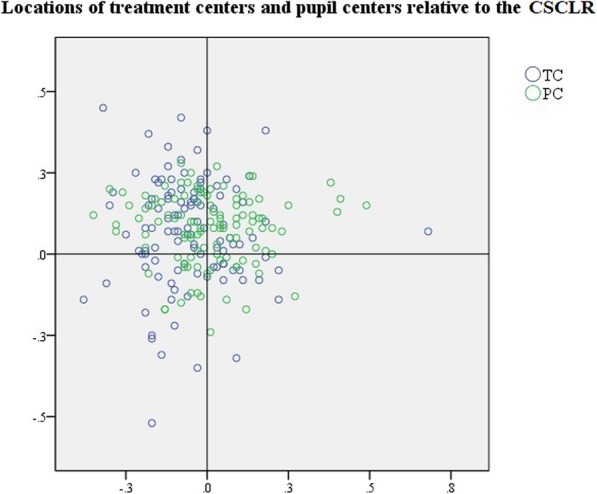
Scatter plot showing the location of treatment centers and pupil centers with respect to CSCLR. TC: treatment center; PC: pupil center

The mean distance of the treatment centers (TC) were 0.20 ± 0.12 mm (range: 0.01–0.58 mm) and 0.20 ± 0.12 mm (range: 0.02–0.56 mm) for the pupil center (PC) and CSCLR, respectively, and showed no significant difference between them (t = 0.32, *P* = 0.75). The mean distance between the preoperative CSCLR and PC (Chord mu) was 0.18 ± 0.09 mm (range: 0.02–0.51 mm). The locations of TC and PC relative to CSCLR were shown in Fig. 2.

**Fig. 3:**
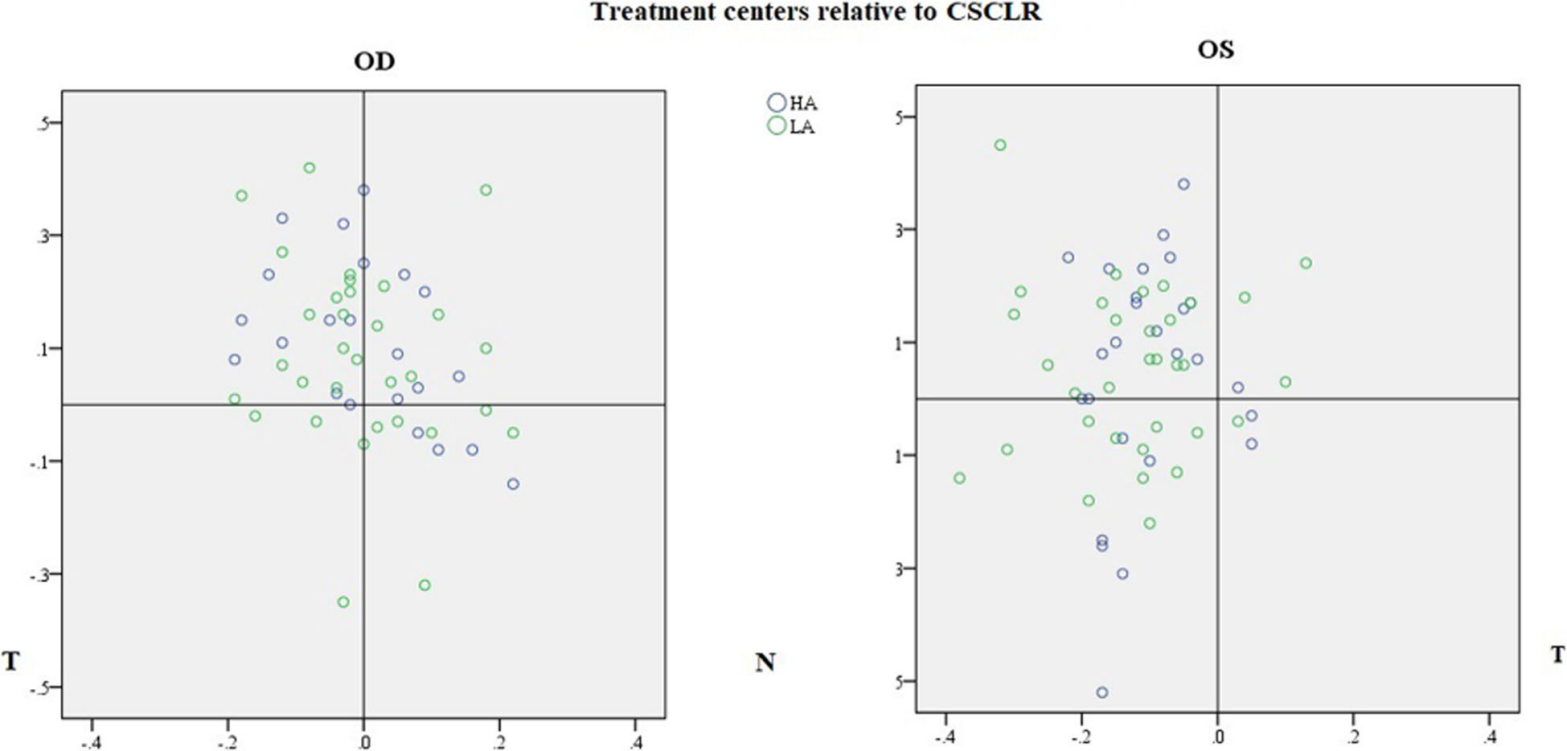
Scatter plot showing the location of treatment centers with respect to CSCLR in the HA and LA groups for the right and left eyes

Figure 3 showed the locations of TC with respect to CSCLR in HA and LA groups for both left and right eyes. No significant difference was found in the decentration distances between HA and LA in both eyes (OD: HA: 0.18 ± 0.10 mm, LA: 0.20 ± 0.14 mm, *P* = 0.659; OS: HA:0.22 ± 0.11 mm, LA:0.20 ± 0.11 mm, *P* = 0.637). No significant difference was found between the two eyes (OD:0.19 ± 0.12 mm, OS:0.21 ± 0.11 mm, *P* = 0.423). In the right eyes, the TC of 40 eyes (72.7%) were superior to CSCLR [SN: 15 (27.3%); ST: 25 (45.5%); IT: 5 (9.1%); IN: 10 (18.2%)], while in the left eyes, the TC of 38 eyes (64.4%) were nasal to the CSCLR [SN: 34 (57.6%); ST: 4 (6.8%); IT: 3 (5.1%); IN: 18 (30.5%)]. No significant difference was found in the probability of distribution in each quadrant between HA and LA groups in both the eyes (OD: Mann-Whitney U, *P* = 0.521; OS: Mann-Whitney U, *P* = 0.604).

### Impact of decentration on visual outcomes

When analyzed across the 3 levels of decentration (< 0.1 mm, 0.1–0.2 mm, and > 0.2 mm), no statistically significant association between decentration and visual outcomes of predictability, efficiency, and safety in both the groups was observed (Tables 1 and 2). There was no significant association between decentration and postoperative refractive astigmatism, CI and IOS in both groups (Tables 1 and 2). In HA group, there was a significant relationship between the magnitude of decentration and the induced coma and SA. Both induced coma and SA was increased with the amount of decentration. Besides, the comparisons between levels 3 (> 0.2 mm) and 2 (0.1~0.2 mm) demonstrated a statistically significant difference in coma only (LSD: *P* = 0.007). In the LA group, there was no significant association between decentration and induced aberrations. There was no significant association between decentration and postoperative MTF cutoff and OSI in either group (Tables 1 and 2).

**Table 1:** Correlation of visual outcomes with decentration from CSCLR in eyes with astigmatism higher than 2.5 diopters

	Decentration, mm			
	< 0.1 (*n* = 10)	0.1~0.2 (*n* = 16)	> 0.2 (*n* = 22)	P
Predictability (*n* = 48)				
SE within 0.5D	9 (90%)	13 (81.3%)	21 (95.5%)	0.429
SE within 1.0D	0	2 (12.5%)	1 (4.5%)	
SE more than 1.0D	1 (10%)	1 (6.3%)	0	
Efficacy				
UDVA 20/20 or better	9 (90%)	13 (81.3%)	16 (72.7%)	0.520
20/25 to 20/40	1 (10%)	3 (18.8%)	6 (27.3%)	
Worse than 20/40	0	0	0	
Efficacy index (Overall = 1.00 ± 0.18)	0.98 ± 0.18	1.00 ± 0.20	1.00 ± 0.17	0.934
Safety				
Improved by at least 1 line	1 (10%)	3 (18.3%)	3 (13.6%)	0.471
No change	8 (80%)	13(81.3%)	15 (68.2%)	
Worsen by at least 1 line	1 (10%)	0	4 (18.2%)	
Safety index (Overall = 0.99 ± 0.11)	0.97 ± 0.09	1.02 ± 0.11	0.97 ± 0.12	0.406
Correction of astigmatism				
Preoperative cylindrical error by MR (D)	−3.23 ± 0.17	− 3.17 ± 0.70	−3.09 ± 0.62	0.84
Postoperative cylindrical error by MR (D)	−0.68 ± 0.21	− 0.44 ± 0.44	−0.61 ± 0.42	0.261
CI	0.87 ± 0.14	0.89 ± 0.14	0.88 ± 0.14	0.969
IOS	0.21 ± 0.06	0.14 ± 0.15	0.20 ± 0.13	0.329
Wavefront and OSI				
△HOA	0.21 ± 0.15	0.18 ± 0.09	0.31 ± 0.22	0.058
△SA	0.01 ± 0.37	0.18 ± 0.13	0.24 ± 0.21	0.042
△Vertical coma	0.14 ± 0.50	0.29 ± 0.46	0.23 ± 0.45	0.727
△Horizontal coma	−0.20 ± 0.36	−0.22 ± 0.28	− 0.16 ± 0.39	0.843
△Coma	−0.06 ± 0.24	−0.08 ± 0.23	0.16 ± 0.30	0.013
MTF _cutoff_ (cpd)	33.3 ± 8.13	36.6 ± 11.5	35.1 ± 10.1	0.731
OSI	1.0 ± 0.66	0.99 ± 0.70	0.91 ± 0.63	0.904
SR	0.17 ± 0.03	0.19 ± 0.05	0.19 ± 0.05	0.460
OV 100%	1.10 ± 0.25	1.22 ± 0.38	1.18 ± 0.34	0.695
OV 20%	0.75 ± 0.19	0.88 ± 0.29	0.84 ± 0.26	0.464
OV 9%	0.42 ± 0.11	0.49 ± 0.15	0.48 ± 0.15	0.429

*SE* spherical equivalent, *CI* correction index, *IOS* index of success, *△HOA* surgical induced change in higher-order aberrations, *△SA* surgical induced change in spherical aberrations, *OSI* objective scatter index

**Table 2:** Correlation of visual outcomes with decentration from CSCLR in eyes with astigmatism less than 1.5 diopters

	Decentration, mm			
	< 0.1 (*n* = 13)	0.1~0.2 (*n* = 25)	> 0.2 (*n* = 28)	P
Predictability				
SE within 0.5D	13 (100%)	25 (100%)	25 (89.3%)	0.371
SE within 1.0D	0	0	2 (7.1%)	
SE more than 1.0D	0	0	1 (3.6%0	
Efficacy				
UDVA 20/20 or better	13 (100%)	25 (100%)	28 (100%)	0.502
20/25 to 20/40	0	0	0	
Worse than 20/40	0	0	0	
Efficacy index (Overall = 1.07 ± 0.11)	1.07 ± 0.11	1.06 ± 0.11	1.07 ± 0.12	0.955
Safety				
CDVA Improved by at least 1 line	0	1 (4%)	2 (7.1%)	0.585
No change	13 (100%)	24 (96%)	26 (91.9%)	
Worsen by at least 1 line	0	0	0	
Safety index (Overall = 0.98 ± 0.12)	0.98 ± 0.10	0.97 ± 0.13	0.99 ± 0.12	0.786
Correction of astigmatism				
Preoperative cylindrical error by MR (D)	−0.56 ± 0.46	−0.77 ± 0.41	−0.48 ± 0.41	0.047
Postoperative cylindrical error by MR (D)	−0.21 ± 0.17	− 0.15 ± 0.22	− 0.26 ± 0.21	0.165
CI	0.78 ± 0.63	1.02 ± 0.52	0.73 ± 0.62	0.194
IOS	0.33 ± 0.41	0.18 ± 0.29	0.41 ± 0.56	0.179
Wavefront and OSI				
△HOA	0.21 ± 0.17	0.23 ± 0.12	0.21 ± 0.17	0.936
△SA	0.33 ± 0.22	0.36 ± 0.24	0.31 ± 0.22	0.714
△Vertical coma	0.07 ± 0.31	0.16 ± 0.26	0.28 ± 0.36	0.116
△Horizontal coma	−0.22 ± 0.23	−0.33 ± 0.28	−0.21 ± 0.25	0.20
△Coma	0.10 ± 0.16	0.15 ± 0.17	0.08 ± 0.21	0.391
MTF _cutoff_ (cpd)	31.7 ± 9.3	32.8 ± 9.8	35.1 ± 7.8	0.448
OSI	1.06 ± 0.58	0.92 ± 0.55	0.79 ± 0.40	0.256
SR	0.17 ± 0.04	0.20 ± 0.12	0.21 ± 0.11	0.44
OV 100%	1.04 ± 0.30	1.10 ± 0.33	1.16 ± 0.26	0.455
OV 20%	0.70 ± 0.22	0.77 ± 0.24	0.82 ± 0.20	0.295
OV 9%	0.41 ± 0.14	0.46 ± 0.15	0.51 ± 0.13	0.081

*SE* spherical equivalent, *CI* correction index, *IOS* index of success, *△HOA* surgical induced change in higher-order aberrations, *△SA* surgical induced change in spherical aberrations, *OSI* Objective scatter index

## Discussion

It is widely accepted that a centration with regard to the visual axis remains the key point in the induction of optimized visual outcomes while maintaining the functional corneal morphology after refractive surgeries. Pande and Hillman [13] found that the CSCLR was the closest measurable point to the visual axis and so should be used for centration. Angle kappa is the angle between the visual axis and the anatomic pupillary axis of the eye due to displacement of the fovea to the pupillary axis. The current study used the term chord mu instead of angle kappa to describe the offset values between the CSCLR and pupil center according to Chang et al. [10]. We found that the CSCLR was superior to the pupil centers in most of the eyes as reported previously [14]. Horizontally, CSCLR was distributed evenly in the nasal and temporal quadrants. We also found that the preoperative chord mu was higher in the eyes with HA. Angle kappa values were reported to be greater in hypermetropes [15] and emmetropes [16], but till date there is no published study that reported the correlation of astigmatism and angle kappa. Studies on children with retinopathy of prematurity (ROP) demonstrated that the corneal curvature was influenced by the maturity of retina [17, 18]. These findings demonstrated a correlation between the displacement of fovea and HA, and more studies are needed to further confirm this.

We also found that 72.7% of the treatment centers were superior to the CSCLR for right eyes and 88.1% of the treatment centers were nasal to the CSCLR for left eyes. Li et al. reported superior displacements of the treatment centers for both eyes [5], and Lazaridis reported nasal displacements of the treatment centers [19], while Liu et al. found an even distribution of the treatment centers. Due to relatively low levels of suction during docking, an involuntary eye drift may occur in the process of lenticular creation. Hence, the patient should be informed to fixate on the green light during the whole process of docking. The mean decentrations for CSCLR and pupillary center were 0.20 mm in the current study, and this was within the range of values of 0.17 to 0.36 mm published in the earlier reports [6–8, 19, 20]. Decentration that do not exceed 0.3 mm is rarely considered to be visually significant [21].

Chan et al. [9] found a significant correlation between anterior keratometric astigmatism and decentration distance, attributing to the mismatched contact surfaces between an astigmatic cornea and the spherical contact glass. To further clarify this issue, the characteristics of lenticular decentration between the two astigmatic groups were compared. The results showed no significant difference in the amount of decentration between high and low astigmatism groups.

In analyzing the possible associations between the magnitudes of decentration and postoperative visual outcomes, no statistically significant associations were observed between the decentration from CSCLR and the outcomes of predictability, efficiency, and safety in both the groups. Decentered treatments are associated with reduced visual acuity, irregular astigmatism, halos, glare, reduced contrast sensitivity, and monocular diplopia in laser in situ keratomileusis (LASIK) [21, 22]. Currently, there are fewer studies that reported the evaluation of SMILE decentration and its effect on visual outcomes. Wong et al. found no statistical difference in postoperative visual acuity among the different pupillary decentration applied eyes, and then the centration was attempted on the pupil center in this study [8]. Li also reported good visual outcomes despite mild decentration [5]. The good visual outcomes following SMILE might be related to the fact that the centration can be maintained throughout the entire laser procedure since the creation of the lenticule is performed under suction [19].

It has been reported that the increasing decentration caused increasing undercorrection of 2nd order sphere and induction of 2nd order astigmatism after photorefractive keratectomy (PRK). But the effect was limited when the decentration was ≤1.0mm [23, 24]. In the current study, there was no significant association between decentration and the correcting efficacy of astigmatism in both the groups, which was in line with the results reported by chan et al. [9]. Therefore, mild decentration after SMILE has little influence on astigmatism correction, either in high or low astigmatic group.

We also found that the induced coma and SA were greater in eyes with greater decentration in HA group, but no significant associations were found in the LA group. Several studies have demonstrated that decentration mainly induced coma and SA [25–27]. This indicated that the magnitude of aberration after refractive surgery increases with attempted correction. Although the SE was similar in the two groups, the lenticule was significantly thicker in the HA group. Further study is warranted to determine the exact relationship between the amount of attempted correction and the magnitude of induced aberrations. Based on these results, we concluded a decentration of > 0.20 mm from the CSCLR results in greater induction of coma, and SA after SMILE in eyes with HA. In addition, our study also supported the idea that decentration has greater influence on coma-inducing effects [5], as the comparisons between levels 3 (> 0.2 mm) and 2 (0.1~0.2 mm) demonstrated a statistically significant difference in coma only.

In addition to the wavefront aberration, the double-pass system was used to assess the objective visual function. There are studies that used this system for assessing the correlation between optical quality and decentration after Orthokeratology [28] and toric IOL implantation [29]. In the current study, we found no significant association between decentration and postoperative MTF cutoff, OSI, SR, and OVs in either group. Therefore, objective quality of vision measurements was consistent with the subjective visual acuity.

The method that used to determine the magnitude of decentration varied among different studies and affected the magnitude of decentration. Liu [6] and Wong [8] evaluated the decentration using intraoperative calibrated video capture images, while the coordinates of the vertex with respect to the pupil center were measured using the wavefront analysis or topography. Li et al. [5] used a postoperative anterior elevation map and the optical zone was defined as the area below the sphere on the postoperative map when the best-fit sphere was set as the same value as preoperative. Also this study reported a mean decentered displacement of 0.17 mm. Lazaradis et al. [19] used an objective measurement method where the optical zone centration was obtained as the maximum value of the pachymetric difference map. Similar method with that of Reinstein [7] and Kang [20] et al. with a tangential curvature difference map was used in our study. The mean offset of 0.20 mm (range: 0.01–0.58 mm) in our study was considered to be in good agreement with Reinstein’s study, but was smaller than that of the Lazaradis’s study (0.315 mm).

## Conclusion

Preoperative chord mu (angle kappa) is higher in eyes with HA. No significant difference was found in the amount of decentration between high and low astigmatic groups. In the right eyes, 72.7% of the treatment centers were superior to the CSCLR and in the left eyes, 88.1% of the treatment centers were nasal to the CSCLR. There were no significant associations between decentration and the outcomes of predictability, efficiency, safety, MTF cutoff, OSI, SR and OVs in both the groups. Decentration > 0.20 mm resulted in greater induction of coma and SA after SMILE in eyes with HA.

## Data Availability

All data generated or analysed during this study are included in this article.

## Abbreviations

BCVA: Best corrected distance visual acuity
Chord mu: the mean distance between the preoperative CSCLR and PC
CI: the correction index
CSCLR: Coaxial sighted corneal light reflex
HA: High astigmatism group
IN: Inferior nasal
IOS: Index of success
IT: Inferior temporal
LA: Low astigmatism group
MTF: Modulation transfer function
OSI: Objective scatter index
OVs: OQAS values
PC: the pupil center
SA: Spherical aberration
SE: Spherical equivalent
SIA: the surgically-induced change in the astigmatism
SMILE: Femtosecond laser small incision lenticule extraction
SN: Superior nasal
SR: Strehl ratio
ST: Superior temporal
TC: the treatment centers
TIA: the target-induced astigmatism

## References

[1] Sekundo W, Kunert KS, Blum M (2011). Small incision corneal refractive surgery using the small incision lenticule extraction (SMILE) procedure for the correction of myopia and myopic astigmatism: results of a 6 month prospective study. Br J Ophthalmol.

[2] Shah R, Shah S, Sengupta S (2011). Results of small incision lenticule extraction: all-in-one femtosecond laser refractive surgery. J Cataract Refract Surg.

[3] Kamiya K, Shimizu K, Igarashi A (2014). Visual and refractive outcomes of femtosecond lenticule extraction and small-incision lenticule extraction for myopia. Am J Ophthalmol.

[4] Kunert KS, Russmann C, Blum M (2013). Vector analysis of myopic astigmatism corrected by femtosecond refractive lenticule extraction. J Cataract Refract Surg.

[5] Li M, Zhao J, Miao H (2014). Mild decentration measured by a scheimpflug camera and its impact on visual quality following SMILE in the early learning curve. Invest Ophthalmol Vis Sci.

[6] Liu M, Sun Y, Wang D (2015). Decentration of optical zone center and its impact on visual outcomes following SMILE. Cornea..

[7] Reinstein D, Gobbe M, Gobbe L (2015). Optical zone centration accuracy using corneal fixation-based SMILE compared to eye tracker-based femtosecond laser-assisted LASIK for myopia. J Refract Surg.

[8] Wong JX, Wong EP, Htoon HM (2017). Intraoperative centration during small incision lenticule extraction (SMILE). Medicine (Baltimore).

[9] Chan TCY, Wan KH, Kang DSY (2019). Effect of corneal curvature on optical zone decentration and its impact on astigmatism and higher-order aberrations in SMILE and LASIK. Graefes Arch Clin Exp Ophthalmol.

[10] Chang DH, Waring GO (2014). The subject-fixated coaxially sighted corneal light reflex: a clinical marker for centration of refractive treatments and devices. Am J Ophthalmol.

[11] Guell JL, Pujol J, Arjona M (2004). Optical Quality Analysis System; Instrument for objective clinical evaluation of ocular optical quality. J Cataract Refract Surg.

[12] Tian M, Miao H, Shen Y (2015). Intra- and intersession repeatability of an optical quality and intraocular scattering measurement system in children. PLoS One.

[13] Pande M, Hillman JS (1993). Optical zone centration in keratorefractive surgery. Entrance pupil center, visual axis, coaxially sighted corneal reflex, or geometric corneal center?. Ophthalmology..

[14] Srivannaboon S, Chotikavanich S (2005). Corneal characteristics in myopic patients. J Med Assoc Thail.

[15] Basmak H, Sahin A, Yildirim N (2007). Measurement of angle kappa with synoptophore and Orbscan II in a normal population. J Refract Surg.

[16] Giovanni F, Siracusano B, Cusmano R (1988). The angle kappa in ametropia. New Trends Ophthalmol.

[17] Liu F, Yang X, Tang A (2018). Association between mode of delivery and astigmatism in preschool children. Acta Ophthalmol.

[18] Davitt BV, Quinn GE, Wallace DK (2011). Astigmatism progression in the early treatment for retinopathy of prematurity study to 6 years of age. Ophthalmology..

[19] Lazaridis A, Droutsas K, Sekundo W (2014). Topographic analysis of the centration of the treatment zone after SMILE for myopia and comparison to FS-LASIK: subjective versus objective alignment. J Refract Surg.

[20] Kang DSY, Lee H, Reinstein DZ (2018). Comparison of the distribution of Lenticule Decentration following SMILE by subjective patient fixation or triple marking centration. J Refract Surg.

[21] Melki SA, Azar DT (2001). LASIK complications: etiology, management, and prevention. Surv Ophthalmol.

[22] Mulhern MG, Foley-Nolan A, O’Keefe M (1997). Topographical analysis of ablation centration after excimer laser photorefractive keratectomy and laser in situ keratomileusis for high myopia. J Cataract Refract Surg.

[23] Bühren J, Yoon G, Kenner S (2007). The effect of optical zone decentration on lower- and higher-order aberrations after photorefractive keratectomy in a cat model. Invest Ophthalmol Vis Sci.

[24] Padmanabhan P, Mrochen M, Viswanathan D (2009). Wavefront aberrations in eyes with decentered ablations. J Cataract Refract Surg.

[25] Moreno-Barriuso E, Lloves JM, Marcos S (2001). Ocular aberrations before and after myopic corneal refractive surgery: LASIK-induced changes measured with laser ray tracing. Invest Ophthalmol Vis Sci.

[26] Mrochen M, Kaemmerer M, Mierdel P (2001). Increased higher-order optical aberrations after laser refractive surgery: a problem of subclinical decentration. J Cataract Refract Surg.

[27] Lee SB, Hwang BS, Lee J (2010). Effects of decentration of photorefractive keratectomy on the induction of higher order wavefront aberrations. J Refract Surg.

[28] Liu G, Chen Z, Xue F (2018). Effects of myopic Orthokeratology on visual performance and optical quality. Eye Contact Lens.

[29] Debois A, Nochez Y, Bezo C (2012). Refractive precision and objective quality of vision after toric lens implantation in cataract surgery. J Fr Ophtalmol.

